# Influence of symbiotic bacteria on the susceptibility of *Plagiodera versicolora* to *Beauveria bassiana* infection

**DOI:** 10.3389/fmicb.2023.1290925

**Published:** 2023-11-03

**Authors:** Mei Liu, Jinli Ding, Min Lu

**Affiliations:** Key Laboratory of Biocatalysis and Enzyme Engineering, School of Life Sciences, Hubei University, Wuhan, China

**Keywords:** 16S rRNA, *Beauveria bassiana*, symbiotic microbiota, interactions, *Plagiodera versicolora*, fungal virulence and pathogenicity

## Abstract

The symbiotic bacterial microbiota of insects has been shown to play essential roles in processes related to physiology, metabolism, and innate immunity. In this study, the symbiotic microbiome of *Plagiodera versicolora* at different developmental stages was analyzed using 16S rRNA high-throughput sequencing. The result showed that symbiotic bacteria community in *P. versicolora* was primarily made up of *Actinobacteriota*, *Proteobacteria*, *Firmicutes*, *Bacteroidota*, and *Dependentiae*. The bacterial composition among different age individuals were highly diverse, while 65 core genera were distributed in all samples which recommend core bacterial microbiome. The 8 species core bacteria were isolated from all samples, and all of them were classified as *Pseudomonas* sp. Among them, five species have been proven to promote the vegetable growth of *Beauveria bassiana*. Moreover, the virulence of *B. bassiana* against nonaxenic larvae exceeded *B. bassiana* against axenic larvae, and the introduction of the *Pseudomonas* sp. to axenic larvae augmented the virulence of fungi. Taken together, our study demonstrates that the symbiotic bacteria of *P. versicolora* are highly dissimilar, and *Pseudomonas* sp. core bacteria can promote host infection by entomopathogenic fungus. This result emphasizes the potential for harnessing these findings in the development of effective pest management strategies.

## 1. Introduction

*P. versicolora* is a leaf-eating pest that is a member of the *Chrysomelidae* family and widely distributed in the world, including Asia, Europe, and North America (Yoneya et al., 2014; Liu et al., 2023). Both the adults and larvae of this pest are known for their preference for feeding on the young leaves of poplar and willow plants, which causes huge economic losses for the forestry industry (Ling et al., 2021; Liu et al., 2021). Using chemical insecticides like phoxim and malathion can effectively lower the population density, but their persistent use inevitably results in undesirable and harmful consequences for the environment, plants, and human health (Wade and Breden, 1986). As a result, the entomopathogenic fungi that have low environmental impact are gradually developing into an effective strategy for *P. versicolora* control (Hou et al., 2023; Tu et al., 2023).

The insect-pathogenic fungus *B. bassiana* (Hypocreales: *Cordycipitaceae*) has the broadest host spectrum among all insect pathogens and serves as a main source of fungal pesticides to combat against wide-spectrum arthropod pests (Xiao et al., 2012; Vivekanandhan et al., 2018, 2020; Ding et al., 2023a). The host arthropod’s pathogenesis and mycosis by *B. bassiana* begins with the adherence of conidia on the epicuticle of the host (Baek et al., 2022; Chavez et al., 2023). Then, infective cells penetrate through the cuticle, proliferate in hemocoel, secrete toxins, and cause the hosts to die (Joop and Vilcinskas, 2016; Perumal et al., 2023a). The fungus undergoes a transformation from pathogenic proliferation to growth on the host cadavers and reproduces conidia to infect new insect hosts (Ding et al., 2023b; Perumal et al., 2023b). During the entire infection cycle, the virulence and growth of *B. bassiana* can be influenced by symbiotic microbiota of the host (Zhang et al., 2013; Xu et al., 2019; Bai et al., 2023). An example is gut associated bacteria *Bacillus* and *Pseudomonas* which, symbiotic with *Blattella Germanics*, significantly inhibit the germination and growth of *B. bassiana* (Zhang et al., 2013).

The insect harbors a variety of bacterial symbionts like other animals (Sittipo et al., 2018; Xiong, 2022). The composition of microbiota in insects is highly variable, which is promoted by different drivers including diet, environment, physiology, and development stage (Malacrinò, 2021; Zhao et al., 2022). But not all of them are affected by external or internal factors. Some bacteria persist in the insect host regardless of the environmental or physiological changes, these bacteria constitute the core microbiome (Zhao et al., 2022). The core microbiomes have a close evolutionary relationship with insects that has been retained, enriched, and inherited through natural selection, and plays an important role in the degradation of host defense substances and defense against pathogenic microorganisms (Douglas, 2015; Singh et al., 2021).

In this study, the symbiotic microbiome of different ages *P. versicolora* was identified through 16 s rRNA high-throughput sequencing and the core microbiome was determined. The phenomenon that symbiotic microbiota affected the fungi virulence was analyzed by removing and reintroducing the core microbiome. Moreover, *Pseudomonas* sp. of the core microbiome promoted fungal growth and virulence. Overall, this research was described as the intricate relationships between the insects, symbiotic microorganisms, and pathogens, and how these interactions can be harnessed for pest management strategies.

## 2. Materials and methods

### 2.1. Strains culture and insect rearing

*B.bassiana* (Bb476) were obtained from the Institute of Ecology, Shandong Academy of Sciences, Jinan, China, and were maintained on Potato dextrose agar (PDA: 0.6% potato starch, 2% dextrose, and 2% agar) at 25°C. Bacteria isolated from *P. versicolora* was cultured in Luria-Bertani medium (LB:1% peptone, 0.5% yeast extra, 1% NaCl and 1.5% agar). *P. versicolora* were collected from Shahu Park in Wuhan, Hubei Province (30.35°N, 114.33°E) following its appearance characteristics, and they were fed the fresh willow leaves and maintained in a room under controlled conditions (temperature 25 ± 2°C, relative humidity 60 ± 5% and 14 h of light) (Wade, 1994). Surface-sterilized eggs were fed on sterile poplar leaves (washed with 75% ethanol for 8 min) to obtain axenic larvae, and axenic larvae were verified to be aseptic through the bacteria isolation experiments (Liu et al., 2023).

### 2.2. 16S rRNA sequencing

The eggs (E), pupae (P), first instar larva (L1), second instar larva (L1), third instar larva (L1), female adults (BF), and male adults (BM) of *P. versicolora* were collected from Shahu Park in Wuhan, Hubei Province (30.35°N, 114.33°E). The high-throughput sequencing through a 16S rRNA gene fragment were used to detected symbiotic bacteria of collected samples (Bai et al., 2023). Female adults (BF), and male adults (BM) were divided into the body and gut for testing. Each sample included eight parallel replicates, and the pupae, first instar larva, second instar larva, third instar larva, female adults, and male adults were used at approximately 5 larvae per replicate, and the pupae were used at approximately 15 pupae per replicate.

TIANamp DNA kit (TIANGEN, China) was used to extract total DNA from all samples according to the instructions. PCR amplification of the V3-V4 region of the 16S rRNA gene was performed using primer pairs 338F (5’-ACTCCTACGGGAGGCAGCA-3′) and 806R (5’-GGACTACHVGGGTWTCTAAT-3′). All samples were performed according to formal experimental conditions. PCR products of the same sample were mixed and tested by 2% agarose gel electrophoresis. The PCR products were recovered by cutting glue using an AxyPrepDNA gel recovery kit (AXYGEN). Finally, the PCR products were sequenced at Illumina NovaSeq 6,000 for high throughput sequencing. All raw sequencing reads in this study have been uploaded to the NCBI Sequence Read Archive (SRA) database with accession numbers from SRR25927065 to SRR25927134 (Bio Project: PRJNA1013480). After sequencing, Fastp (v0.19.6) was used to control the quality of the raw sequences, Flash (v1.2.7) was used to splice the double-ended sequences, and DADA2 was used to diagnose the valid tags, to obtain the initial ASV (amplicon sequence variant) for species annotation. Microbial diversity and community composition were analyzed using the vegan package in R (version 3.3.1).

Rarefaction curves constructed using Mothur (version 1.30) based on α diversity indices were used to illustrate whether the amount of sequencing data for the samples was reasonable. The α diversity index of the community (includes Chao, Shannon, Simpson, and Ace) can reflect the sequencing depth, richness, and evenness of the community. The Kruskal-Wallis test was used to compare the α diversity index of different samples. β diversity was evaluated by using dimensionality reduction analysis based on the weighted unifrac distance algorithm. In this study, principal coordinate analysis (PCoA) and non-metric multidimensional scaling (NMDS) were used to compare β diversity among different groups based on Adonis. Hierarchical clustering analysis was performed based on the beta diversity distance matrix, and the UPGMA algorithm was used to construct a tree structure to visualize the different degrees of community distribution in different environmental samples. R (version 3.3.1) and Python (version 2.7) were used to draw heat maps of all samples. The heatmap represents the size of data in tables by color gradients and presents information about community species composition.

### 2.3. Infection bioassays

To examine fungal virulence, the axenic and nonaxenic larvae of *P. versicolora* were used as the bioassay hosts, and each assay included three parallel replicates, using approximately 35 larvae per replicate (Liu et al., 2023). Fungal strains were cultured on PDA plates for 10 d at 25°C, and the resultant conidia were used as infectious inocula. The mycelia and conidia were harvested from the plate and suspended in 0.02% Tween 80 solution, followed by violent votexing. The resultant mixture was filtered through the cotton column, and the filtrated conidia was used to infect the hosts. For infection bioassays, the insects were immersed in conidial suspension (1 × 10^8^ cells/mL) for 30 s and the daily mortality was recorded, and then the log-rank test was used to determine the statistical differences between the paired curves.

### 2.4. Measurement of fungi vegetative growth and virulence

The core bacteria isolated from *P. versicolora* were cultured in 50 mL liquid Luria-Bertani medium (1% peptone, 0.5% yeast extra and 1% NaCl) at 28°C. After 24 h, the fermentation of core bacteria was collected and filtered through the microporous membrane (0.22 μm in pore size). 2 × PDA culture medium (1.2% potato starch, 4% dextrose, and 4% agar) added to the fermentation suspension in a 1:1 ratio as FB medium was used to measure vegetative growth of fungi, and PDA culture medium as a control. 5 mm hypha colonies were inoculated in different FB mediums at 25°C for 10 days, and the colony diameters were measured.

To investigate how the core microbiome affects the infection virulence of *B. bassiana*, the axenic larvae reintroduced to the core microbiome were used as the bioassay hosts to test fungi virulence. The core microbiome reintroduction was performed as described previously (Demirci et al., 2013). Axenic larvae were fed with leaves of willow, the surface of which were coated by the core bacteria, and reintroduced larvae were verified through the bacteria isolation experiments. After that, the same pathogenic fungal infection method was used for biometric tests, and mortality was observed and recorded.

### 2.5. Statistical analysis

Prior to statistical analysis, the Kolmogorov–Smirnov test and Levene test were performed to test the normality and homogeneity of all variances, respectively (Ding et al., 2023a,b). The Kaplan–Meier method was used to analyze the survival curves, and the log-rank test was used to evaluate the significance of differences.

## 3. Result

### 3.1. Analysis of the symbiotic bacteria richness and diversity between different age larvae

The symbiotic bacteria richness and diversity between different age *P. versicolora* were analyzed. A total of 1,592,856 valid sequences of symbiotic bacteria were obtained from 72 samples. Based on a 97% similarity level of classification and an average sequencing coverage of 99.88%, 7,879 bacterial ASVs (Amplicon sequence variants) were found, belonging to 38 phyla and 1,038 genera, respectively (Supplementary Table S1). The rarefaction curve was increased with the increase of sequencing depth and this indicated that the measured data could meet the subsequent experimental analysis (Supplementary Figure S1).

**Figure 1:**
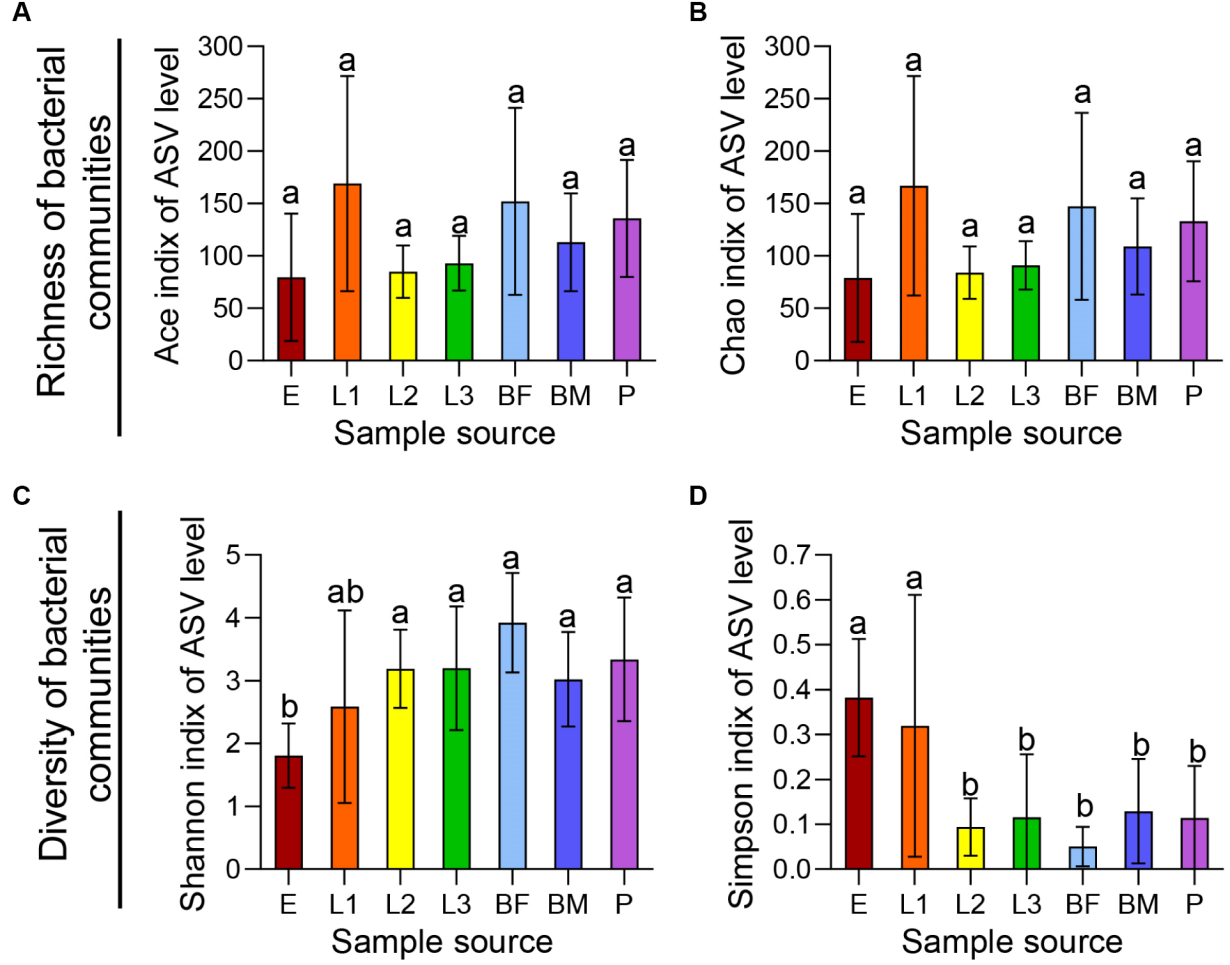
Richness and diversity of symbiotic bacteria in different age *P. versicolora*. The eggs (E), pupae (P), first instar larva (L1), second instar larva (L2), third instar larva (L3), female adults (BF), and male adults (BM) of *P. versicolora* were collected from Shahu Park in Wuhan, Hubei Province (30.35°N, 114.33°E). The α diversity of symbiotic bacteria in all samples were shown through alpha diversity analysis. **(A)** ACE index. **(B)** Chao index. **(C)** Shannon index. **(D)** Simpson index.

**Figure 2:**
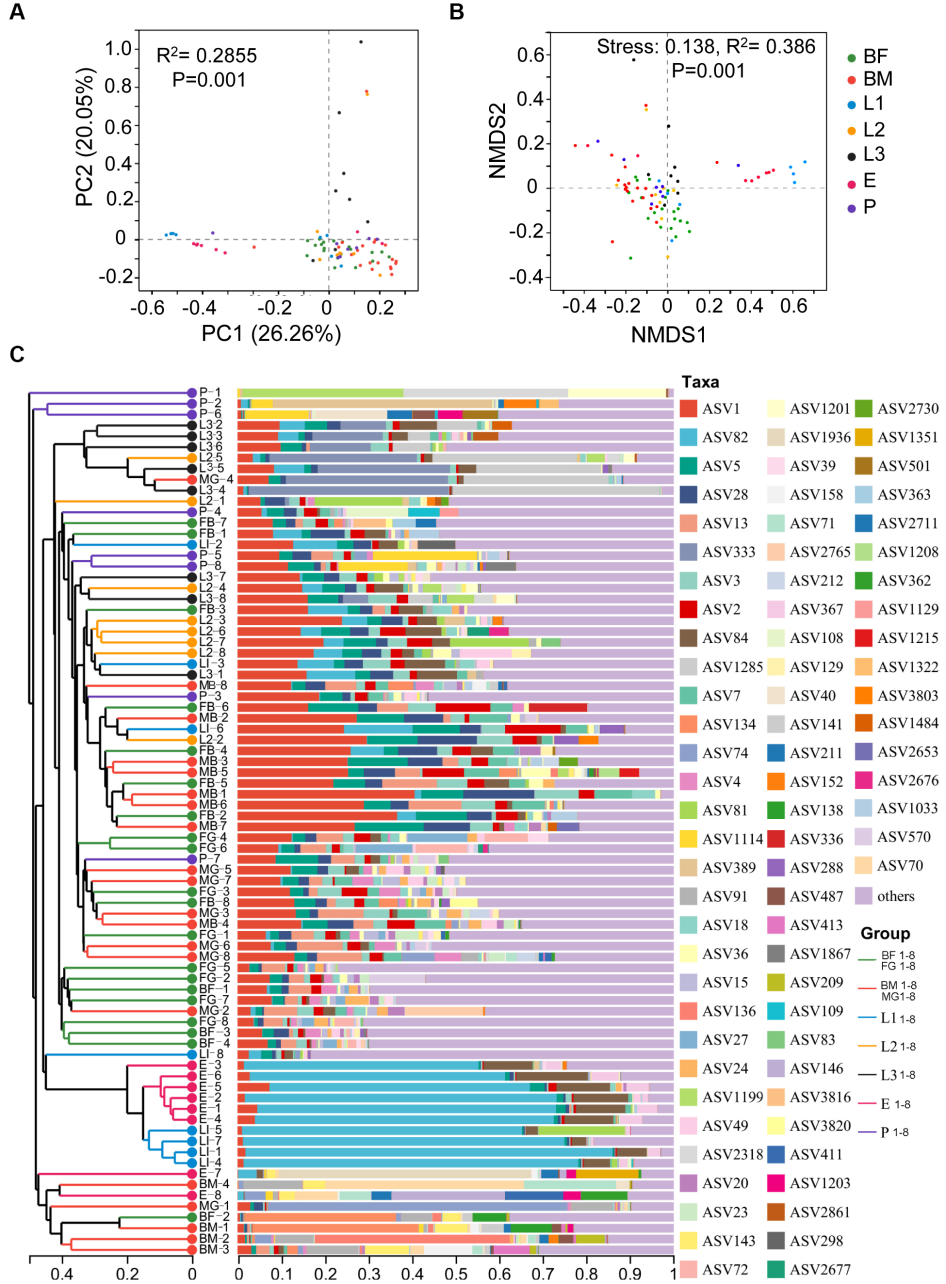
The composition and diversity of bacterial microbiota in different age larvae. **(A)** Principal Coordinate analysis (PCoA) and **(B)** Nonmetric Multidimensional Scaling (NMDS) analysis was showed the diversity of symbiotic bacteria in different age larvae based on the weighted UniFrac metric. **(C)** Community analysis of symbiotic bacteria in different age larvae.

Alpha diversity analysis based on ASV level is shown in Figure 1, The ACE index and Chao index reflected community species richness, and there was no significant difference in microbial community richness among 7 sets of samples (Figures 1A,B). Shannon index and Simpson index indicated community species diversity and showed significant differences among texted samples (Figures 1C,D). β-diversity analysis of all samples based on ASV levels was shown in Figure 2. PCoA (Adonis, *p* < 0.001) and NMDS (Adonis, stress = 0.138; *p* < 0.001) analyses based on the weighted UniFrac metric were showing significant differences, this revealed that microbial community composition among the seven groups were significantly different (Figures 2A,B). The Hierarchical clustering analysis based on the bray-Curtis distance metric reflected that seven groups had higher diversity and abundance of the bacterial microbiota (Figure 2C).

**Figure 3:**
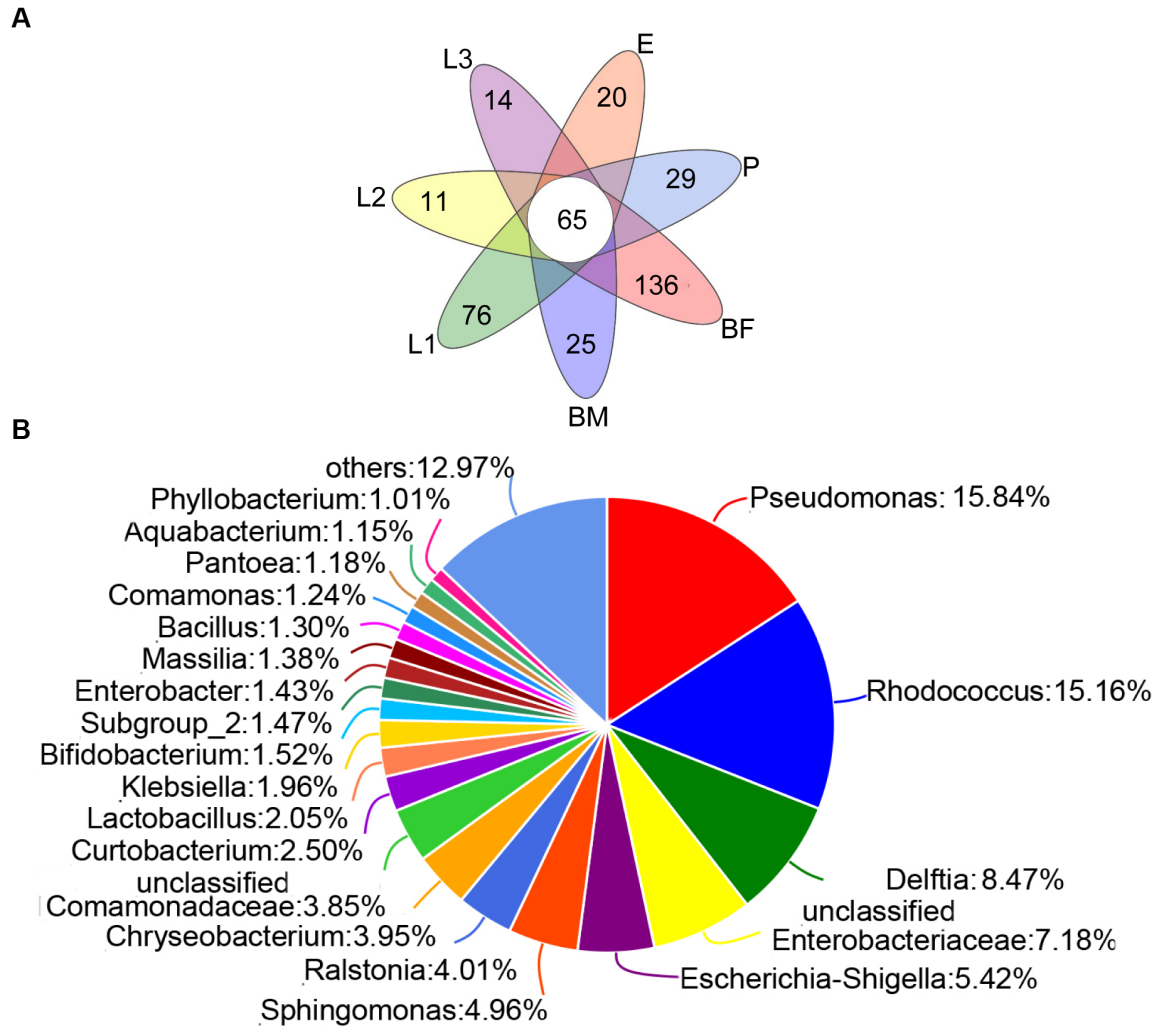
The composition of corn bacterial microbiota in *P. versicolora*. **(A)** Venn diagram was displayed the genus number of symbiotic bacteria in different age larvae. **(B)** A pie chart of common species for corn bacterial microbiota was displayed at the genus level.

**Figure 4:**
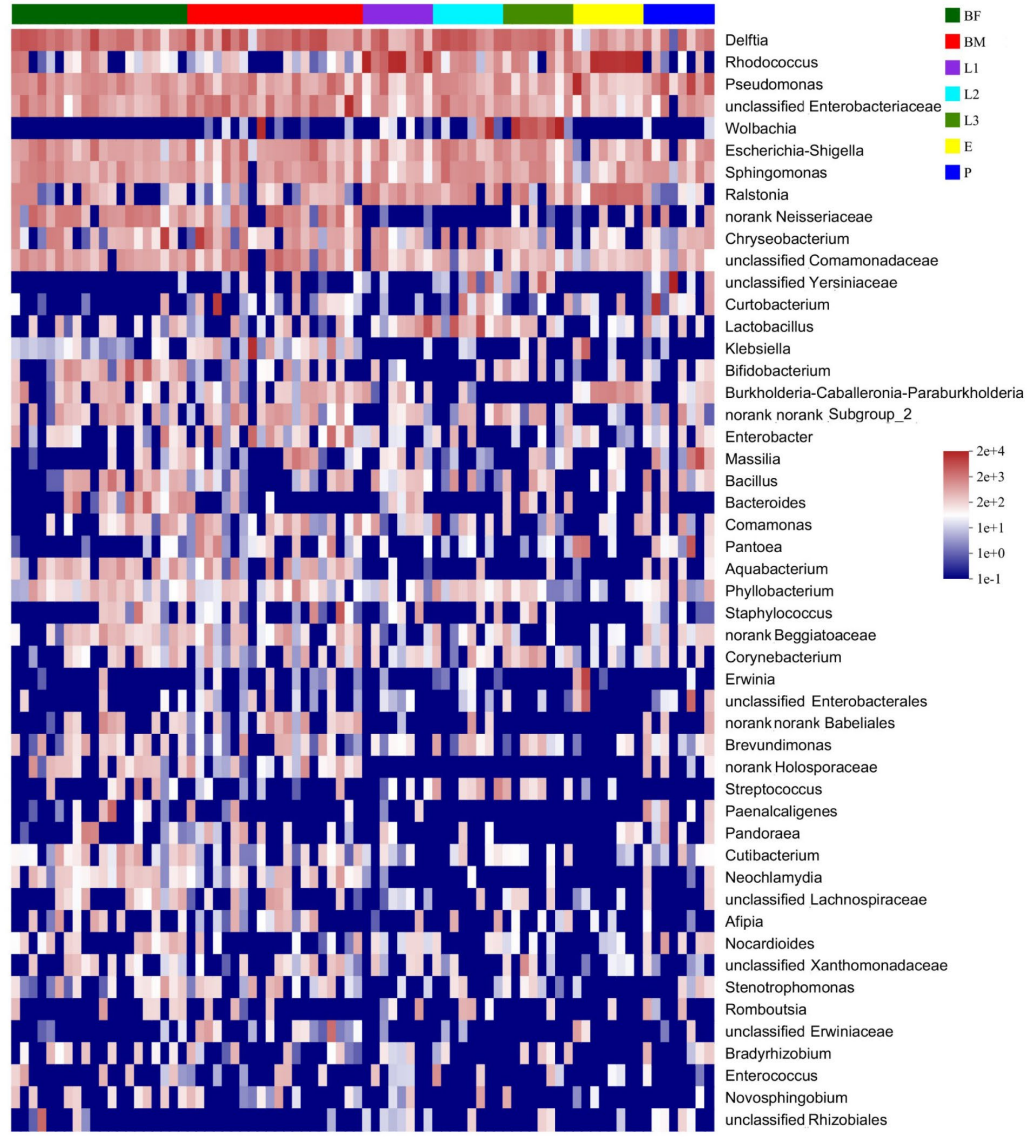
Heatmap of the top 50 genera abundances of corn bacterial microbiota was displayed at the genus classification level.

As shown in the Figure 3A, 25 unique genera were found in the BM group, and 163, 76, 11, 14, 20, and 29 unique genera were distributed in BF, L1, L2, L3, E, and P groups respectively, and the unique bacteria species of different sample were listed in Supplementary Table S2. Among those, 65 genera were distributed in all samples, which were named the core microbiome. Out of these 65 genera, 15.84% belong to *Pseudomonas*, 15.16% belong to *Rhodococcus*, and 8.74% belong to *Delftia* (Figure 3B). The community heat map showed that 65 core bacteria belong to 50 genera, with *Delftia* sp.*, Pseudomonas* sp., *Rhodococcus* sp., and *Enterobacteriaceae* sp. as the common genera of all samples (Figure 4).

### 3.2. Symbiotic bacteria accelerated the mortality of *Plagiodera versicolora* larvae infected by *Beauveria bassiana*

**Figure 5:**
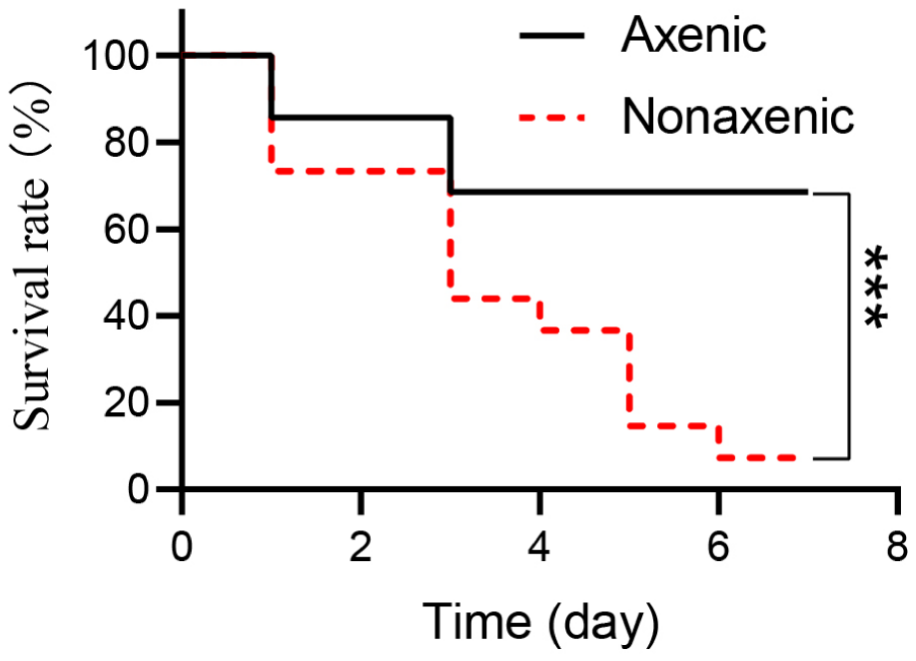
The mortality of axenic and nonaxenic *P. versicolora* larvae larvae infected by *B. bassiana*. Fungal strains were incubated on PDA plates for 10 days at 25°C, and the resultant conidia were used as infectious propagules. In bioassay, the insect hosts were immersed in conidial suspension (10^8^ conidia/ml). The resultant insects were reared at 25°C, and accumulative mortality was recorded daily. Survival percentage was plotted against the time post-infection, and statistical difference between the paired curves was analyzed by log rank test.

To evaluate the role of symbiotic bacteria in the host interplay with pathogenic microorganisms, axenic and nonaxenic larvae were used to test host susceptibility to *B. bassiana*. Interestingly, at 6 days post-infection, the mortality of nonaxenic larvae infected by *B. bassiana* was significantly higher than those of axenic larvae (Figure 5, Log-rank test, *p* < 0.001). These results indicated that symbiotic bacteria accelerated the mortality of *P. versicolora* larvae infected by *B. bassiana*.

### 3.3. *Pseudomonas* sp. promote fungi vegetative growth

**Figure 6:**
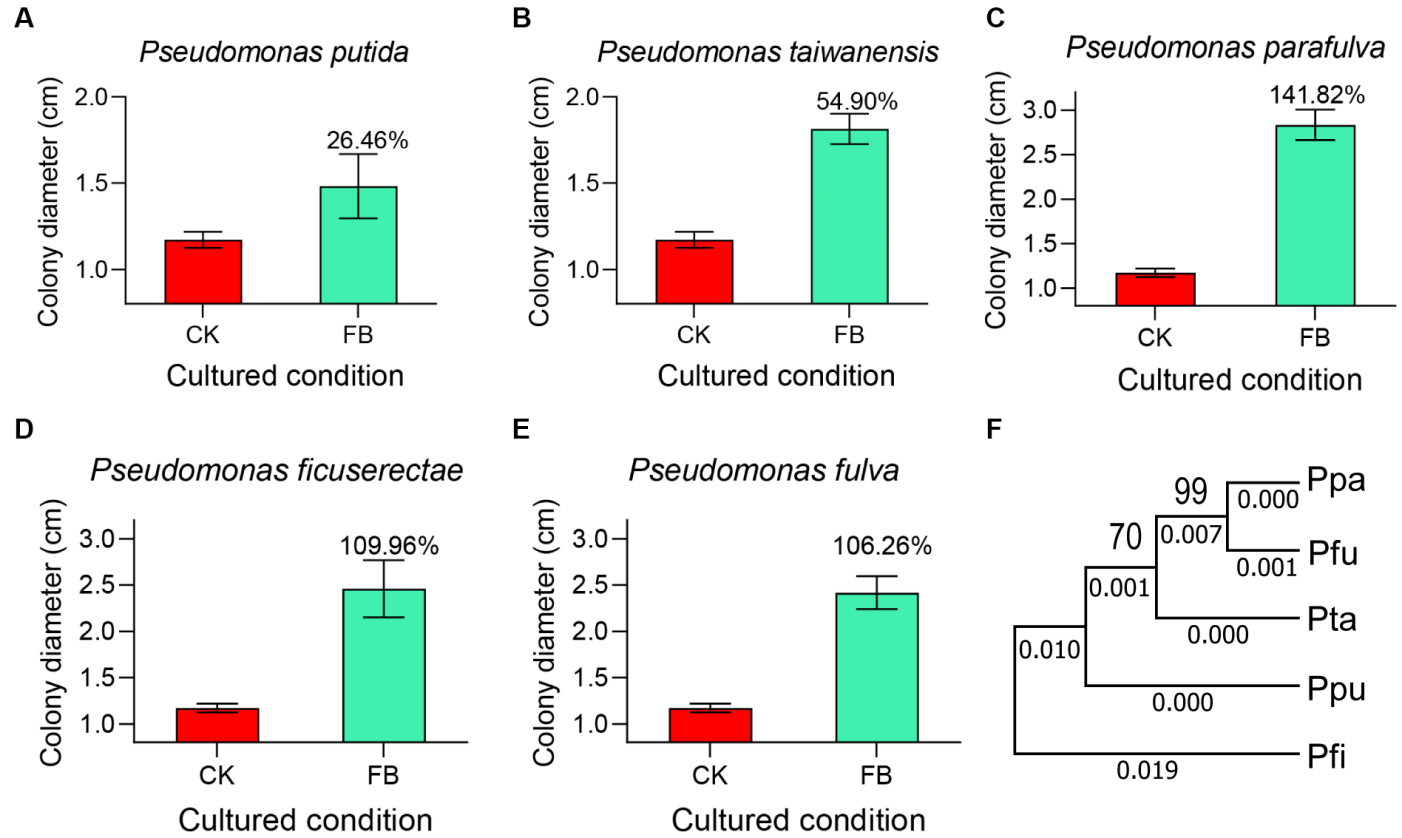
The core microbiome affects the mycelium growth. Fungal strains were cultured on PDA plates and different FB mediums for the condition. After 7 days of incubation at 25°C, colony morphologies were recorded. **(A)** Fermentation broth medium of *Pseudomonas putida*. **(B)** Fermentation broth medium of *Pseudomonas taiwanensis*. **(C)** Fermentation broth medium of *Pseudomonas parafulva*. **(D)** Fermentation broth medium of *Pseudomonas ficuserectae*. **(E)** Fermentation broth medium of *Pseudomonas fulva*. **(F)** The evolutionary relationship of five core strains was analyzed through MEGA7. Ppa represents *Pseudomonas parafulva*; Pfu represents *Pseudomonas fulva*; Pta represents *Pseudomonas taiwanensis*; Ppu represents *Pseudomonas putida*; Pfi represents *Pseudomonas ficuserectae*.

Out of 65 core microbial genera, only 8 species were obtained through the bacteria isolation experiments, and they are *Pseudomonas putida*, *Pseudomonas taiwanensis*, *Pseudomonas parafulva*, *Pseudomonas ficuserectae*, *Pseudomonas fulva*, *Pseudomonas meliae*, *Pseudomona aeruginosa*, and *Pseudomonas punonensis, respectively.* To investigate whether the core microbiome plays an important role in *P. versicolora* susceptibility to *B. bassiana*, fungi vegetative growth on the fermentation medium was detected. As shown in Figure 6, on the PDA medium, the colony diameter of *B.bassiana* was 1.25 ± 0.11 cm. On the added *P.putida*, *P. taiwanensis*, *P. parafulva*, *P. ficuserectae*, and *P. fulva* fermentation media, the colony diameter increased by approximately 26.46, 54.90, 141.82, 109.96 and 106.26%, respectively. These five strains are highly conserved in evolution and belong to *Pseudomonas* sp. (Figure 6F). However, there was no significant difference in the colony diameter between the *P.meliae*, *P.aeruginosa*, and *P.punonensis* fermentation media and PDA medium. The results showed that *Pseudomonas* sp. Played an important role in the growth rate of *B. bassiana*.

### 3.4. The isolated *Pseudomonas* sp. enhanced the pathogenesis of *Beauveria bassiana* against *Plagiodera versicolora*

**Figure 7:**
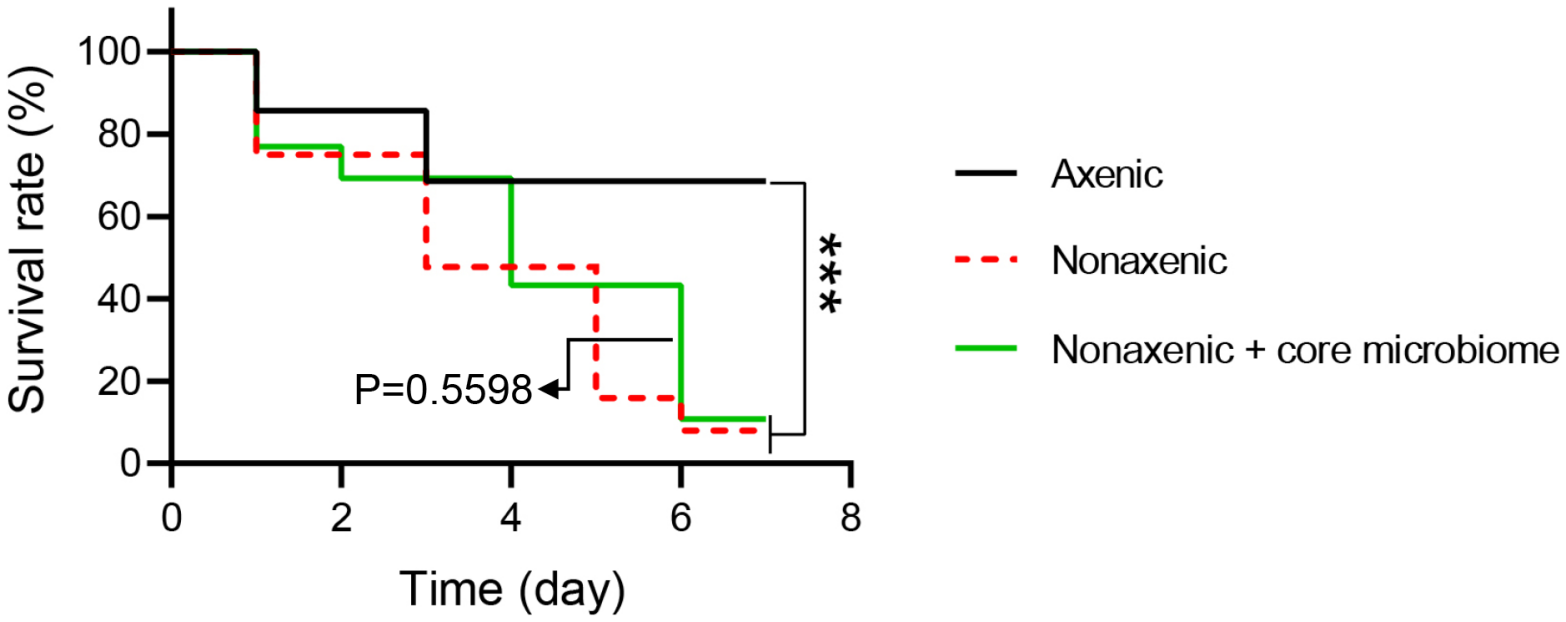
The survival rate of axenic, nonaxenic and bacteria reintroduced *P. versicolora* larvae infected by *B. bassiana*. The log-rank test was used to evaluate the significance of differences among groups, ***p < 0.001.

To refine the specific functions of the core microbiome on *B. bassiana* pathogenesis to *P. versicolora* larvae, *P.putida*, *P. taiwanensis*, *P. parafulva*, *P. ficuserectae*, and *P. fulva* were reintroduced into axenic larvae. All the bacteria reintroduced into the larvae significantly accelerated the mortality of larvae at 4 to 6 d post-*B. bassiana* infection. There is a significant difference between the mortality of the reintroduced core microbiome group and the axenic group (Log-rank test, *p* < 0.001) and no significant difference between the reintroduced core microbiome group and the nonaxenic group (Log-rank test, *p* = 0.5598). These results suggest that the core microbiome of *Pseudomonas* sp. promotes *B. bassiana* pathogenesis to *P. versicolora* (Figure 7).

## 4. Discussion

In this study, 16 s rRNA high-throughput sequencing was used to analyze the diversity and variability of symbiotic bacteria in different age *P. versicolora* larvae. Taxonomic analysis revealed that the symbiotic bacteria community of *P. versicolora* was mainly composed of *Actinobacteriota*, *Proteobacteria*, *Firmicutes*, *Bacteroidota*, and *Dependentiae*. Similar results were also reported in other Coleoptera, such as *Gastrolina depressa, Dendroctonus valens* and *Dendroctonus rhizophagus* (Morales-Jimenez et al., 2012; Xu et al., 2019; Ma et al., 2021). The environment where insects live or the diet that insects feed upon significantly affects the structure of their symbiotic bacteria (Malacrinò, 2021; Zhao et al., 2022). *Rahnella* sp.*, Micrococcus luteus* and *Pantoea agglomerans* were present in a large proportion of lab-reared *P.versicolora* larvae, but in very small quantities in wild-collected larvae (Demirci et al., 2013). Nevertheless, *Sphingomonas* sp. was detected in all the samples in both wild-collected *P. versicolora*, which suggested that there was a core community of bacteria (Demirci et al., 2013). Various studies also indicated that the persistence of some core symbiotic bacterial microbiota occurs regardless of the diet and environment in insect species (Turnbaugh et al., 2009; Xu et al., 2019; Ma et al., 2021). Those symbiotic bacteria play an important ecological role in maintaining the population of insects (Turnbaugh et al., 2009). Analysis of symbiotic bacteria of different ages of *P. versicolora* shows that there are 65 types of symbiotic bacteria present in the all sample, mainly *Pseudomonas*, *Rhodococcus*, and *Delftia* et al. Among them, *Pseudomonas* is a genus of gram-negative bacteria able to colonize a wide range of niches and is also a core microbiome in humans, soil, and plants (Turnbaugh et al., 2009; Chevalier et al., 2017; Hong et al., 2023).

Bacteria not only act as symbionts but also as pathogens of eukaryotes (mammals, insects, and plants). Their ability to enter and inhabit eukaryotic tissues/cells has given bacteria specialized niches that are stable, nutrient-rich, and enemy-free (Waters and Bassler, 2005). When hosts obtain benefits from bacteria or no negative impact (such as improving the insect’s fitness, or intervening in host-pathogen interactions), the relationship is referred to as symbiosis (Malacrinò, 2021; Zhao et al., 2022). On the other hand, bacteria that cause damage to hosts are called pathogens (Demirci et al., 2013). After the insects were infected by entomopathogenic fungi, the symbiotic bacteria in the host will transform into pathogenic bacteria. An example is *Metarhizium rileyi* infected insects, in which the gut symbiotic bacteria are translocated to hemolymph, its host humoral antibacterial immunity is activated, and fungi infection is enabled (Wang et al., 2023). Our study showed that symbiotic bacteria significantly enhanced the pathogenesis of *B. bassiana* against *P. versicolora*. Notably, the virulence of *B. bassiana* against nonaxenic larvae exceeded *B. bassiana* against axenic larvae, and the introduction of the core microbiome to axenic larvae augmented the virulence of fungi. The introduction of core microbiota significantly enhances fungal virulence. A large part of this was caused by the bacterial enzymes, such as proteases (alkaline metalloproteases) and chitinases which were identified as key contributors to tissue degradation and fungal defense system suppression (Ansari et al., 2005; Pham et al., 2007; Cirimotich et al., 2011). The larvae of *Dendroctonus Valens LeConte* infection by *B. bassiana* induced gut microbiota dysbiosis, such as *Erwinia* sp., which expedited entomopathogenic fungal infection (Xu et al., 2019). In other insects, symbiotic bacteria that affect *B. bassiana* virulence has also been reported. For example, symbiotic bacteria (*Pseudomonas* sp., *Enterobacter* sp., and *Microbacterium* sp.) of *Lymantria dispar* promoted the infection virulence of *B. bassiana* (Bai et al., 2023). Analogous outcomes were evident in mosquito research (Davis et al., 2019). Other studies have pointed out that *Aphids* harboring endosymbiont *Regiella insecticola* exhibited heightened resistance to entomophthorales fungus (Sanda et al., 2023). This underscores the multifaceted role of symbiotic bacteria of insects in modulating *B. bassiana* infectivity. The symbiotic microbiome also demonstrated a nutritionally reciprocal relationship with *B. bassiana*. The fermented liquid of core symbiotic bacteria was enhanced *B. bassiana* growth rates.

In summary, this study investigated the composition of symbiotic microbiomes of different ages of *P. versicolora* and identified the core microbiome. In addition, the core microbiome of *Pseudomonas* sp. was proven to promote the fungal infection process in *P. versicolora*. These findings provide fundamental insights into the multilateral interactions among insect hosts, communal microbiota, and entomopathogenic fungus, which may ultimately lead to a better strategy for biological control of *P. versicolora*.

## Data availability statement

The datasets presented in this study can be found in online repositories. The names of the repository/repositories and accession number(s) can be found at: https://www.ncbi.nlm.nih.gov/, PRJNA1013480.

## Ethics statement

The manuscript presents research on animals that do not require ethical approval for their study.

## Author contributions

MLi: Formal analysis, Investigation, Visualization, Writing – original draft. JD: Formal analysis, Investigation, Writing – review & editing. MLu: Conceptualization, Funding acquisition, Project administration, Supervision, Writing – review & editing.
