# Supplementary material for: Influence of symbiotic bacteria on the susceptibility of Plagiodera versicolora to Beauveria bassiana infection
Source: Front Microbiol. 2023 Nov 3;14:1290925. doi: 10.3389/fmicb.2023.1290925 (PMC10655113; doi:10.3389/fmicb.2023.1290925)
Supplement: Supplementary file 2 [file Data_Sheet_2.docx]

Supplementary Material

# Supplementary Figures and Tables

For more information on Supplementary Material and for details on the different file types accepted, please see [here](https://www.frontiersin.org/guidelines/author-guidelines#supplementary-material).

## Supplementary Figures

**
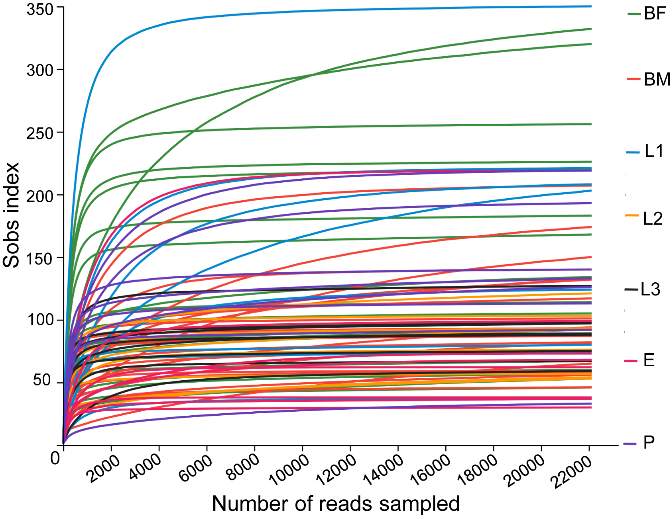
**

**Supplementary Figure 1.** Rank abundance curves of all samples based on ASVs.


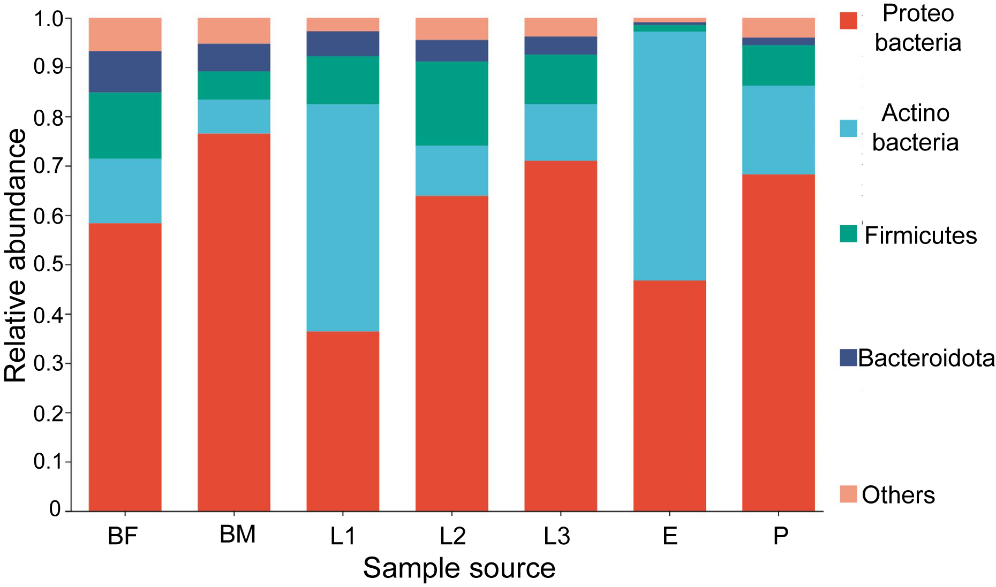


**Supplementary Figure 2.** The relative abundance of symbiotic bacteria at the phylum level in *P. versicolora*.

## Supplementary Tables

| **Table S2. Detailed information for each part of the Venn diagram.** | |
| --- | --- |
|  | |
| Part | Genus |
| 20 genera unique to egg | g__Vulgatibacter  g__Dyella  g__norank_f__Sandaracinaceae  g__Mogibacterium  g__norank_f__Bacteroidales_RF16_group  g__unclassified_f__Burkholderiaceae  g__Chthonobacter  g__norank_f__norank_o__norank_c__Subgroup_11  g__Frondihabitans  g__Pseudenhygromyxa  g__unclassified_f__Longimicrobiaceae  g__Brooklawnia  g__norank_f__p-2534-18B5_gut_group  g__Spirosoma  g__norank_f__Paludibacteraceae  g__unclassified_f__Ilumatobacteraceae  g__Muribaculum  g__Pseudorhodoferax  g__Chloronema  g__Ferrovibrio |
| 29 genera unique to pupa | g__Roseisolibacter  g__Belnapia  g__Plantibacter  g__unclassified_o__Polyangiales  g__Duganella  g__unclassified_f__Fimbriimonadaceae  g__norank_f__Bacillaceae  g__Natronincola  g__Brevibacillus  g__norank_f__norank_o__norank_c__norank_p__SAR324_cladeMarine_group_B  g__Pseudactinotalea  g__Legionella  g__Aquipuribacter  g__Ornithinibacter  g__Angustibacter  g__Candidatus_Methylopumilus  g__norank_f__Atopobiaceae  g__Luteibacter  g__Rubritepida  g__Psychrobacter  g__Candidatus_Riegeria  g__Alcaligenes  g__Anaerocolumna  g__norank_f__norank_o__norank_c__Actinobacteria  g__unclassified_f__Nocardiaceae  g__Thermobacillus  g__Agrococcus  g__Clostridium_sensu_stricto_3  g__Criblamydia |
| 163 genera unique to female | g__Advenella  g__Neisseria  g__norank_f__cvE6  g__Candidatus_Paracaedibacter  g__Sphingorhabdus  g__Cellulosilyticum  g__Pir4_lineage  g__Solibacillus  g__unclassified_f__Nitrincolaceae  g__norank_f__norank_o__norank_c__AKAU4049  g__Salinicola  g__Paenochrobactrum  g__unclassified_p__Gemmatimonadota  g__Pseudoclavibacter  g__Eubacterium_nodatum_group  g__Chujaibacter  g__Knoellia  g__Granulicatella  g__norank_f__Microtrichaceae  g__Lutispora  g__Hydrogenoanaerobacterium  g__norank_f__Carnobacteriaceae  g__Blvii28_wastewater-sludge_group  g__Georgfuchsia  g__Hungatella  g__Catellatospora  g__Frankia  g__norank_f__norank_o__Sva1033  g__Thermoactinomyces  g__Ureibacillus  g__unclassified_f__Paenibacillaceae  g__unclassified_o__Gaiellales  g__Capnocytophaga  g__unclassified_f__Dermabacteraceae  g__Paeniglutamicibacter  g__Candidatus_Protochlamydia  g__norank_f__Ilumatobacteraceae  g__Polyangium  g__unclassified_p__Chloroflexi  g__unclassified_f__Pseudomonadaceae  g__Limibaculum  g__norank_f__Vermiphilaceae  g__Erythrobacter  g__OLB12  g__Thermoanaerobacter  g__Clostridium_sensu_stricto_18  g__Clostridium_sensu_stricto_15  g__norank_f__Lachnospiraceae  g__Amnipila  g__Dechlorobacter  g__Chryseolinea  g__norank_f__Peptococcaceae  g__unclassified_c__Bacteroidia  g__unclassified_o__Bacillales  g__norank_f__Sutterellaceae  g__Actinoallomurus  g__unclassified_f__Microtrichaceae  g__Keratinibaculum  g__hgcI_clade  g__Fastidiosipila  g__unclassified_f__Nitrosococcaceae  g__Niveispirillum  g__norank_f__norank_o__S085  g__norank_f__A0839  g__Butyrivibrio  g__Methyloceanibacter  g__Sediminibacterium  g__unclassified_p__Myxococcota  g__Alloprevotella  g__Castellaniella  g__unclassified_f__Solimonadaceae  g__Dysgonomonas  g__Roseibacterium  g__Adlercreutzia  g__norank_f__Oscillospiraceae  g__norank_f__SM2D12  g__unclassified_f__Methyloligellaceae  g__Phaselicystis  g__norank_f__Paenibacillaceae  g__Hathewaya  g__norank_f__Rhodobacteraceae  g__norank_f__norank_o__Rhizobiales  g__norank_f__Entotheonellaceae  g__norank_f__Dethiobacteraceae  g__Chitinophaga  g__Aurantimonas  g__Procabacter  g__Defluviicoccus  g__Longispora  g__norank_f__Pleomorphomonadaceae  g__Odoribacter  g__norank_f__norank_o__norank_c__Thermoleophilia  g__norank_f__norank_o__Blfdi19  g__Herpetosiphon  g__Thermopolyspora  g__Defluviimonas  g__Rikenella  g__unclassified_o__Bacteroidales  g__Cupriavidus  g__Proteus  g__norank_f__Myxococcaceae  g__Desulfitobacterium  g__Gulosibacter  g__norank_f__norank_o__Thalassobaculales  g__mle1-7  g__norank_f__norank_o__SAR202_clade  g__norank_f__Intrasporangiaceae  g__Saccharomonospora  g__norank_f__norank_o__norank_c__D8A-2  g__norank_f__norank_o__Peptostreptococcales-Tissierellales  g__Lachnospiraceae_AC2044_group  g__BBMC-4  g__Haloactinopolyspora  g__norank_f__norank_o__norank_c__Lineage_IIb  g__norank_f__A21b  g__Sphingosinicella  g__Clostridium_sensu_stricto_10  g__Lautropia  g__norank_f__norank_o__PLTA13  g__norank_f__Marinococcaceae  g__Oceanobacillus  g__Telmatospirillum  g__Lachnospiraceae_UCG-004  g__possible_genus_06  g__Anaerostipes  g__Tumebacillus  g__unclassified_o__Peptostreptococcales-Tissierellales  g__unclassified_f__Desulfovibrionaceae  g__Haloimpatiens  g__Nitrosomonas  g__Methylocella  g__Arsenicitalea  g__Sarcina  g__unclassified_f__Staphylococcaceae  g__norank_f__Rs-E47_termite_group  g__NK4A214_group  g__norank_f__norank_o__Bradymonadales  g__Enteractinococcus  g__unclassified_f__Pasteurellaceae  g__norank_f__norank_o__norank_c__bacteriap25  g__norank_f__norank_o__Ga0077536  g__Gallicola  g__FFCH7168  g__Planktosalinus  g__Lachnospiraceae_FCS020_group  g__norank_f__Silvanigrellaceae  g__Eubacterium_hallii_group  g__Microbispora  g__Domibacillus  g__Kytococcus  g__norank_f__norank_o__KIST-JJY010  g__Azospira  g__norank_f__norank_o__norank_c__norank_p__RCP2-54  g__Actinobacillus  g__Thermincola  g__Proteiniclasticum  g__norank_f__norank_o__norank_c__BD2-11_terrestrial_group  g__Treponema  g__Nitrococcus  g__norank_f__norank_o__Azospirillales  g__norank_f__37-13  g__Prevotellaceae_UCG-001  g__norank_f__Bacteroidetes_BD2-2 |
| 25 genera unique to male | g__Negativicoccus  g__unclassified_f__Myxococcaceae  g__unclassified_f__Chitinophagaceae  g__Janibacter  g__Desulfitibacter  g__norank_f__norank_o__JG36-TzT-191  g__Candidatus_Finniella  g__Ellin517  g__Pseudochrobactrum  g__Alicycliphilus  g__Longimicrobium  g__C1-B045  g__norank_f__norank_o__norank_c__norank_p__FCPU426  g__norank_f__norank_o__JG36-GS-52  g__Rhizorhapis  g__Thermomonas  g__28-YEA-48  g__Thiopseudomonas  g__unclassified_p__Firmicutes  g__Paludibacter  g__Zoogloea  g__Lentimicrobium  g__Nordella  g__norank_f__norank_o__norank_c__SJA-28  g__unclassified_f__Saprospiraceae |
| 76 genera unique to L1 | g__Kaistia  g__Aetherobacter  g__unclassified_o__Syntrophales  g__Ktedonobacter  g__Pseudoxanthomonas  g__unclassified_o__Acidobacteriales  g__unclassified_f__Sphingobacteriaceae  g__Caldisericum  g__unclassified_o__Streptosporangiales  g__norank_f__PHOS-HE36  g__norank_f__Euzebyaceae  g__IS-44  g__Acidicaldus  g__norank_f__Prolixibacteraceae  g__Megasphaera  g__Tardiphaga  g__Sphaerochaeta  g__norank_f__Hungateiclostridiaceae  g__Rhodanobacter  g__Tyzzerella  g__Halomonas  g__norank_f__Sporichthyaceae  g__norank_f__norank_o__norank_c__Pla4_lineage  g__Dermatophilus  g__norank_f__Dysgonomonadaceae  g__norank_f__Hydrogenophilaceae  g__Tetragenococcus  g__norank_f__norank_o__norank_c__Subgroup_5  g__Fonticella  g__norank_f__norank_o__SJA-15  g__Azoarcus  g__norank_f__norank_o__Actinomarinales  g__norank_f__Moraxellaceae  g__Nocardia  g__norank_f__Rhodocyclaceae  g__Ruminofilibacter  g__Oxobacter  g__Marivivens  g__norank_f__Solirubrobacteraceae  g__Candidatus_Competibacter  g__Nonlabens  g__Cetobacterium  g__Lachnotalea  g__Rahnella1  g__Terrabacter  g__Methylobacter  g__Syntrophorhabdus  g__Candidatus_Actinomarina  g__ADurb.Bin063-1  g__norank_f__norank_o__211ds20  g__Tolumonas  g__Thermoflavimicrobium  g__unclassified_o__Desulfitobacteriales  g__Providencia  g__Methylorosula  g__Cellulosimicrobium  g__Lacibacter  g__norank_f__unclassified  g__norank_f__norank_o__Elev-16S-1166  g__Streptosporangium  g__Chelatococcus  g__unclassified_f__Paludibacteraceae  g__Paludicola  g__Iodobacter  g__unclassified_o__Babeliales  g__norank_f__norank_o__Run-SP154  g__Haematobacter  g__Candidatus_Caldatribacterium  g__Pseudorhizobium  g__Ahniella  g__norank_f__Weeksellaceae  g__norank_f__norank_o__R7C24  g__unclassified_f__Acidobacteriaceae_Subgroup_1  g__Starkeya  g__Cronobacter  g__norank_f__Rhizobiales_Incertae_Sedis |
| 11 genera unique to L2 | g__Kingella  g__Erysipelotrichaceae_UCG-003  g__Paraeggerthella  g__norank_f__Aerococcaceae  g__Hymenobacter  g__Anaerovorax  g__unclassified_f__Dysgonomonadaceae  g__Yonghaparkia  g__norank_f__KD3-93  g__S5-A14a  g__Luedemannella |
| 14 genera unique to L3 | g__Smaragdicoccus  g__Macellibacteroides  g__unclassified_f__Desulfuromonadaceae  g__Enterorhabdus  g__UCG-003  g__Nocardiopsis  g__Dialister  g__norank_f__Halobacteroidaceae  g__alphaI_cluster  g__Bilophila  g__unclassified_f__Cellulomonadaceae  g__unclassified_f__Streptomycetaceae  g__norank_f__norank_o__C0119  g__unclassified_f__Nannocystaceae |
| 65 genera shared by all ages | g__unclassified_f__Rhizobiaceae  g__Novosphingobium  g__Stenotrophomonas  g__Pseudomonas  g__Ralstonia  g__Delftia  g__Chryseobacterium  g__Comamonas  g__norank_f__Beggiatoaceae  g__Rhodococcus  g__Atopostipes  g__unclassified_f__Enterobacteriaceae  g__Bacillus  g__unclassified_o__Chlamydiales  g__Enterococcus  g__Methylobacterium-Methylorubrum  g__Shewanella  g__Blautia  g__Nocardioides  g__Microbacterium  g__Blastococcus  g__Phyllobacterium  g__Flaviflexus  g__Brevundimonas  g__Pantoea  g__Shinella  g__Massilia  g__norank_f__norank_o__Frankiales  g__Turicibacter  g__Cutibacterium  g__Acinetobacter  g__unclassified_f__Comamonadaceae  g__Aquabacterium  g__Paracoccus  g__Lactobacillus  g__Enterobacter  g__Dietzia  g__unclassified_f__Xanthomonadaceae  g__Klebsiella  g__Curtobacterium  g__Bifidobacterium  g__Terrisporobacter  g__norank_f__norank_o__Alteromonadales  g__Escherichia-Shigella  g__Faecalibacterium  g__Gemmobacter  g__Devosia  g__Pseudogracilibacillus  g__unclassified_f__Sphingomonadaceae  g__Frigoribacterium  g__Peredibacter  g__Deinococcus  g__P3OB-42  g__Geobacillus  g__Romboutsia  g__Rubellimicrobium  g__unclassified_f__Lachnospiraceae  g__unclassified_o__Rhizobiales  g__Sphingomonas  g__norank_f__norank_o__Subgroup_2  g__Corynebacterium  g__Sphingopyxis  g__Afipia  g__Mycobacterium  g__unclassified_o__Enterobacterales |
